# A systematic review of double-bundle versus single-bundle posterior cruciate ligament reconstruction

**DOI:** 10.1186/s12891-016-0896-z

**Published:** 2016-01-27

**Authors:** Yan-Song Qi, Hai-Jun Wang, Shao-Jie Wang, Zheng-Zheng Zhang, Ai-Bing Huang, Jia-Kuo Yu

**Affiliations:** Institute of Sports Medicine, Peking University Third Hospital, No. 49, North Garden Road, Haidian District, Beijing, China

## Abstract

**Background:**

Posterior Cruciate Ligament (PCL) ruptures are common sports injuries. One of the key controversies in PCL reconstruction is whether double-bundle reconstruction provides biomechanical and clinical outcomes superior to single-bundle reconstruction.

**Methods:**

We performed a comprehensive search in multiple databases to evaluate the advantages of single-bundle or double bundle reconstructions in anteroposterior stability, graft tension, rotational stability, and functional outcome.

**Results:**

Biomechanical comparisons evaluating anteroposterior stability described either no difference or increased stability in double-bundle reconstructions. Comparing these results is complicated by different graft choices, tensioning techniques, and tunnel positions. Biomechanical studies of graft tension demonstrated conflicting results regarding the optimal reconstruction technique. Seven retrospective clinical studies of single- and double-bundle reconstructions with methodological limitations reported no difference in clinical outcome.

**Conclusions:**

The superiority of single-bundle or double-bundle posterior cruciate ligament reconstruction remains uncertain.

## Background

As the primary restraint to posterior translation in the uninjured knee [1, 2], the posterior cruciate ligament (PCL), which largely consists of the anterolateral (AL) and posteromedial (PM) bundles, is the strongest ligament in the knee joint. Despite initial reports of good functional results with nonoperative treatment of PCL injuries, additional biomechanical and clinical studies suggest a less benign natural history of PCL deficiency resulting in persistent symptoms and premature osteoarthritis [3–7]. Longer-term follow-up studies have also described an increased incidence of arthritis and declining knee function, making PCL reconstruction more widely accepted, especially as operating techniques improving [4, 5, 8, 9]. Although operative indications for these injuries remain controversial, there remains a strong interest in the literature regarding methods of reconstruction [10–12].

Both concerns for premature arthritic change and persistent instability as well as in vitro studies showing restoration of knee biomechanics motivated attempts at surgical reconstruction of the PCL. There is some controversy over the single bundle (SB) versus double bundle (DB) PCL reconstruction methods [13]. Many specialists have recently reported that DB reconstruction is useful in restoring knee functions to an intact knee [14–17], but these conclusions were based mainly on experimental studies that did not investigate the biological healing process. In addition, other studies have shown that the SB technique is effective at reconstructing the AL bundle [18–20], making it difficult to distinguish the advantages of either technique.

These two reconstructions have been reviewed previously [13]. However, these studies were published more than five years and are not comprehensive because many kinematic and clinical documents comparing single-bundle and double-bundle PCL reconstructions have been published in recent years. We therefore performed a systematic review to evaluate the biomechanical and clinical literature on single-bundle versus double-bundle PCL reconstruction and to define which method of reconstruction is superior. We hypothesized that double-bundle PCL reconstruction provides biomechanical and clinical outcomes superior to single-bundle reconstruction.

## Methods

**Fig. 1:**
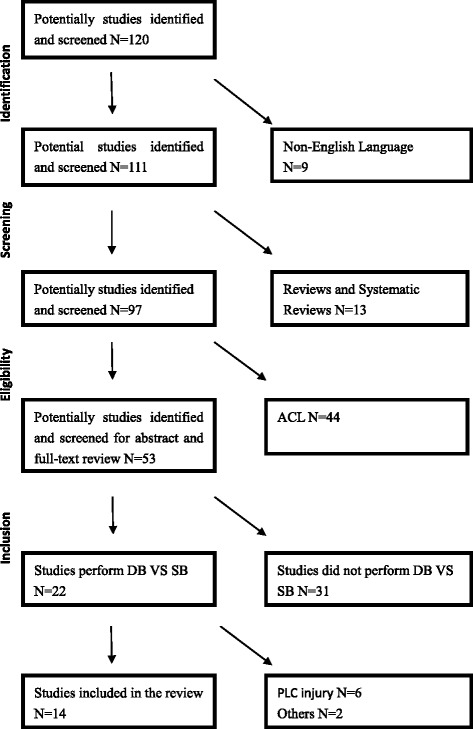
Literatures screening process

October 1, 2014, we used the PubMed, OVID and EMBASE databases to conduct a systematic review of the available English language literature according to PRISMA standards [21, 22] and a PRISMA checklist (Table 1). Initial search words were based on “single bundle AND double bundle AND posterior cruciate ligament reconstruction”. Overall, 120 publications were identified. Both print journals and e-published journals were eligible for inclusion whereas reviews were not eligible. Studies of only one type of PCL reconstruction and non–English language studies were excluded. PLC(posterolateral corner) injury was not included in our study, because the mechanism of injury, pathology, and the function of PLC was quite different. All references from the included literature were checked to assess for articles missed by the initial search criteria (Fig. 1).

We identified five clinical comparison studies and six biomechanical comparisons that evaluated reconstructions for isolated PCL injuries. Review of the references from these studies identified one additional biomechanical and two clinical comparisons. The biomechanical studies are reviewed first, followed by the clinical studies in order of the publication date (Table 2).

**Table 1:** PRISMA checklist

Section/Topic	#	Checklist item	Reported on page#
Title			
Title	1	Identify the report as a systematic review, meta-analysis, or both.	1
Abstract			
Structured summary	2	Provide a structured summary including, as applicable: background; objectives; data sources; study eligibility criteria, participants, and interventions; study appraisal and synthesis methods; results; limitations; conclusions and implications of key findings; systematic review registration number.	2
Introduction			
Rationale	3	Describe the rationale for the review in the context of what is already known.	3-4
Objectives	4	Provide an explicit statement of questions being addressed with reference to participants, interventions, comparisons, outcomes, and study design.	4
Methods			
Protocol and registration	5	Indicate if a review protocol exists, if and where it can be accessed [e.g., Web address], and, if available, provide registration information including registration number.	
Eligibility criteria	6	Specify study characteristics [e.g., PICOS, length of follow-up] and report characteristics [e.g., years considered, language, publication status] used as criteria for eligibility, giving rationale.	4
Information sources	7	Describe all information sources [e.g., databases with dates of coverage, contact with study authors to identify additional studies] in the search and date last searched.	4
Search	8	Present full electronic search strategy for at least one database, including any limits used, such that it could be repeated.	4
Study selection	9	State the process for selecting studies [i.e., screening, eligibility, included in systematic review, and, if applicable, included in the meta-analysis].	4-5 and Figure 1
Data collection process	10	Describe method of data extraction from reports [e.g., piloted forms, independently, in duplicate] and any processes for obtaining and confirming data from investigators.	4-5
Data items	11	List and define all variables for which data were sought [e.g., PICOS, funding sources] and any assumptions and simplifications made.	4,18
Risk of bias in individual studies	12	Describe methods used for assessing risk of bias of individual studies [including specification of whether this was done at the study or outcome level], and how this information is to be used in any data synthesis.	
Summary measures	13	State the principal summary measures [e.g., risk ratio, difference in means].	
Synthesis of results	14	Describe the methods of handling data and combining results of studies, if done, including measures of consistency [e.g., I2] for each meta-analysis.	4-5
Risk of bias across studies	15	Specify any assessment of risk of bias that may affect the cumulative evidence [e.g., publication bias, selective reporting].	
Additional analyses	16	Describe methods of additional analyses [e.g., sensitivity or subgroup analyses, meta-regression].	
Results			
Study selection	17	Give numbers of studies screened, assessed for eligibility, and included in the review, with reasons for exclusions at each stage, ideally with a flow diagram.	4-5 and Figure 1
Study characteristics	18	For each study, present characteristics for which data were extracted [e.g., study size, PICOS, follow-up period] and provide the citations.	5-12
			19-24
Risk of bias within studies	19	Present data on risk of bias of each study and, if available, any outcome level assessment [see item 12].	
Results of individual studies	20	For all outcomes considered [benefits or harms], present, for each study: [a] simple summary data for each intervention group [b] effect estimates and confidence intervals, ideally with a forest plot.	25-26
			Table 2 and Table 3
Synthesis of results	21	Present results of each meta-analysis done, including confidence intervals and measures of consistency.	
Risk of bias across studies	22	Present results of any assessment of risk of bias across studies [see Item 15].	
Additional analysis	23	Give results of additional analyses, if done [e.g., sensitivity or subgroup analyses, meta-regression [see Item 16]].	
Discussion			
Summary of evidence	24	Summarize the main findings including the strength of evidence for each main outcome; consider their relevance to key groups [e.g., healthcare providers, users, and policy makers].	13-16
Limitations	25	Discuss limitations at study and outcome level [e.g., risk of bias], and at review-level [e.g., incomplete retrieval of identified research, reporting bias].	16-17
Conclusions	26	Provide a general interpretation of the results in the context of other evidence, and implications for future research.	17
Funding			
Funding	27	Describe sources of funding for the systematic review and other support; role of funders for the systematic review.	18

## Results

### Biomechanical studies

In 1998, Race and Amis [16] reported the first biomechanical comparison of isometric, single-bundle, and double bundle PCL reconstructions. In this study, they constructed eight cadaveric knees following PCL deficiency models and reconstructed using isometric, single-bundle, or double-bundle techniques. The AL single bundle reconstructions restored anteroposterior (AP) stability from 0° to 60°but showed laxity at higher degrees of flexion. The double-bundle reconstructions, in contrast, restored AP laxity from 0° to 120° to within 1 mm of the intact specimens. They recommended the double-bundle PCL reconstruction technique for patients requiring stability at high flexion angles such as snowboarders and windsurfers [23].

Harner et al. [14] published another biomechanical analysis of single- and double-bundle transtibial PCL reconstructions in 10 cadaveric knees, comparing intact, deficient, and reconstructed PCL specimens from full extension to 120° of flexion under posterior tibial translational load. They demonstrated that, compared to the intact knee, single-bundle reconstructions had poor performance in posterior tibial translation at all flexion angles. The double-bundle reconstructions could reproduce the graft tension at 0° and 30° of flexion and produce in situ tension closer to the intact knee, which support the application of the double-bundle reconstruction.

Wijdicks CA et al. [24] used 18 match-paired cadaveric knees to evaluate the kinematics of a sectioned PCL using single-bundle and double-bundle reconstructions. At ≥ 15°, particularly at 105° of flexion, the double-bundle reconstructions had significantly lower posterior translation than the single-bundle reconstructions. At ≥ 90°, the double-bundle reconstructions had a significantly greater rotational stability than the single-bundle reconstructions. The authors concluded that double bundle PCL reconstructions more closely approximated native knee kinematics than single bundle PCL reconstructions.

Mannor et al. [15] tried to change the position of the femoral attachment site to conduct a biomechanical comparison of single and double-bundle PCL reconstructions in 12 cadaveric knees. The study compared femoral attachments in 3 positions(high-shallow, mid-shallow, mid-deep) using single-bundle reconstructions with the AL bundle in the high-shallow position and double-bundle reconstructions with the PM bundle in either the mid-shallow or mid-deep position. Relative to the intact knees, the single-bundle reconstructions with high-shallow and mid-deep femoral insertions had increased average posterior tibial translation; By contrary, the average posterior tibial translation of reconstructions with the mid-shallow femoral insertion site did not differ from that of intact specimens except at 60° of flexion. The shallow insertion grafts developed greater tension in flexion, but the deep grafts had higher tension in extension. The shallow-shallow double-bundle reconstructions had nearly the same average posterior translation as the intact knees, whereas shallow-deep reconstructions had little posterior translation. Interestingly, both bundles developed increasing tension in flexion in the shallow-shallow configuration, whereas the shallow-deep configuration developed reciprocal tension. The data show that two types of double-bundle reconstructions and the single-bundle shallow reconstructions could reproduce posterior stability to within 2 mm of the intact knees. The authors suggested that it was better to choose a single-bundle reconstruction with a shallow femoral insertion to reproduce AP translation, and the clinical failure of single-bundle reconstruction may be attributed to graft elongation resulting from high graft tension.

Using a tibial inlay technique, Bergfeld et al. [25] further investigated single- and double-bundle reconstructions (performed in random order) in eight cadaveric knees. They used a 100-N posterior translational force to measure the AP translation at 10°, 30°, 60°, and 90° of flexion. Without rotational stability testing, the authors reported no significant difference in AP stability between the intact state and either type of reconstruction.

Markolf et al. [26] evaluated the knee kinematics of single- and double-bundle PCL reconstructions performed on cadaveric knees,which also used a tibial inlay technique. The PM portion was performed with either a narrow or wide bridge from the AL bundle and was tensioned at both 10 and 30 N at 30° of flexion. The AL bundles were tensioned with the strengh determined by restoration of AP laxity to within 1 mm of the intact knees (35.2 ± 10.2 N) at 90°. This study showed that the single AL bundle restored laxity to the intact knee at 45°, 70°, and 90° but enhanced laxity at 0°, 10°, and 30°. The wide-bridge PM bundle tensioned at 10 N significantly lowered AP laxity compared with the single-bundle reconstruction, which had no significant difference in laxity from the intact knees. However, tensioning at 30 N overconstrained the knee at 10°. The narrow-bridge reconstructions were comparable to the wide-bridge reconstructions. The data also showed the mean graft forces in the DB reconstructions were significantly higher, particularly in knee extension. While this study suggested that the addition of a PM bundle at the expense of increased graft forces resulted in a small (1 to 2 mm) but statistically significant improvement in laxity with, there is no clinical evidence. In 2010, Markolf et al. [27] supported their research about the biomechanical properties in 10 fresh-frozen cadaveric knee specimens of three reconstructions: a single AL graft, and AL and PM grafts placed in divergent femoral tunnels with or without a 3-mm bone bridge. Anterior-posterior laxity was adjusted with the intact PCL at 0 °, 10° , 30° , 45° , 70° , and 90° by a ±100 N tibial force. Mean laxities with a single bundle were within 1.2 mm of normal, between 0° and 90°, and means with double-bundle grafts were 1.7 mm to 2.4 mm less than normal, between 10 ° and 45° . The length change of the AL graft from 0 °–90 ° was within 11.3 mm, and the PM graft placed in either tunnel tightened approximately 6 mm with a knee extension from 90 to 0°. Mean forces with a single bundle did not significantly differ from PCL forces for any loading mode tested at 0°; and for which the with double-bundle grafts were 74 N to 154 N higher. Relative to intact knees, the double-bundle reconstruction externally rotated the tibia between 0° and 50° during passive knee extension. Between narrow and wide tunnel separations, there were no significant differences in any biomechanical property. The authors were uncertain whether adding a PM bundle is worth the increased operative demands.

### Clinical studies

In 2004, the first clinical study addressing this controversy was published by Wang et al. [28], who prospectively evaluated 35 patients randomly assigned to single-bundle or double-bundle PCL reconstruction for a minimum of 2 years. A total of 19 single-bundle reconstructions and 16 double-bundle reconstructions were performed. Indications for surgery were the failure of rehabilitation for a minimum of 3 months with persistent functional disability and instability. Combined ligamentous injury and PCL avulsion fractures were precluded. Reconstructions were performed with autologous hamstrings (double or triple semitendinosus/gracilis) with a transtibial tunnel technique. The AL and PM bundles were, respectively, tensioned at 90° and 20° of flexion. There is no significant difference in functional scores, ligament laxity, patient satisfaction, or radiographic examination between the single- and double-bundle reconstructions after a minimum 2-year follow-up. The result showed that approximately 25 % of the patients had residual mild to moderate posterior ligament laxity (<10 mm). None had residual grade III laxity; however, individual improvements in laxity were not reported. The incidence of degenerative changes for both reconstructions were approximately the same (31 % ~ 32 %).

Houe and Jorgensen [29] reconstructed sixteen patients with PCL instability for 6 months and a posterior laxity greater than 10 mm, using single- and double-bundle methods. The arthroscopic single-bundle reconstructions were performed with patellar tendon autograft and semitendinosus/gracilisautograft was used for the double-bundle reconstructions. The AL bundles were tensioned at 70° of flexion during the operation, whereas the PM bundles were tensioned with the maximal manual pull at 20°. With the exception of a median decrease in AP laxity at 30° and 70° of 3 mm, there was no significant difference in laxity or Lysholm score between the 2 types of reconstruction (2-year follow-up, at least).

In both 2008 and 2012, Fanelli et al. [30, 31] published abstracts comparing the clinical results of 45 single-bundle PCL reconstructions and 45 double-bundle PCL reconstructions using allograft tissue (level V evidence). All reconstructions were performed with the transtibial tunnel technique using fresh frozen allograft tissue from the same tissue bank. Achilles tendon and tibialis anterior allograft were used for the AL and PM bundles, respectively. The knees were evaluated postoperatively, comparing the single- to the double-bundle results, with KT-1000 arthrometer (Medmetric Corporation, San Diego, CA) testing, three different knee ligament rating scales(Lysholm, Tegner, and Hospital for Special Surgery knee ligament rating scales) and Telos stress radiography (Marburg, Germany; Austin Associates, Fallston, MD). The authors concluded that neither surgical procedure was clearly superior.

Kim et al. [32] evaluated 21 patients treated with 3 types of PCL reconstructions and followed up for a minimum of 2 years (Therapeutic Level III). A total of 8 patients were treated with a transtibial single-bundle procedure, and the others were performed with transtibial inlay technology (11, single-bundle; 10, double-bundle). An Achilles tendon allograft was used in each case. The data showed that there is no significant difference between two single-bundle reconstructions for posterior tibial translation, whereas the arthroscopic double-bundle group significantly reduced the AP laxity to the transtibial single-bundle group. The mean range of motion and Lysholm scores were similar among the three groups. The authors suggested that, despite its technical difficulty, the arthroscopic tibial inlay double-bundle technique is the best choice to reconstruct the PCL.

Shon OJ et al. [33] evaluated the clinical outcomes of arthroscopically assisted single and double bundle tibial inlay reconstructions of an isolated posterior cruciate ligament injury. Overall, 14 patients underwent single-bundle reconstruction, and 16 patients underwent double-bundle reconstruction used tibial inlay technology. The mean follow-up periods for the two groups were 90.5 months and 64 months, respectively. Lysholm knee scores and stress radiography using a Telos device were performed, and there was no significant difference between the two groups.

Yoon KH et al. [2] analysed 53 cases of two reconstructions (25,SB;28,DB) using Achilles tendon allograft with a minimum 2-year follow-up. The side-by-side difference in posterior translation significantly improved in the two groups, but there was no preoperative difference in posterior instability between the groups except at the final follow-up. The DB reconstruction for PCL ruptures using the Achilles allograft showed better results in posterior stability and IKDC knee examination form than the SB reconstruction did. It is hard to say that DB reconstruction is superior to SB reconstruction clinically and functionally, because there was no difference in their subjective scores, although the 1.4 mm difference in posterior stability was statistically significant.

Li Y et al. [34] published an article in 2014 to verify whether posterior cruciate ligament reconstruction with the double-bundle technique improved stability of the knee relative to the single-bundle technique. This prospective study included 50 patients who were randomised to undergo PCL reconstruction using tibialis anterior grafts with one of the two techniques (25:25) and a minimum of 2 years follow-up. Overall, three patients in the SB group and one patient in the DB group failed in the follow-up. No differences were found between the two groups regarding patient demographic data and the duration from injury to operation (*P* > .05). The Lysholm score, the Tegner activity score and the side-by-side difference in posterior translation were not significantly different between the two groups. According to the International Knee Documentation Committee (both objective and subjective), the DB group had a better grade distribution and a statistically higher grade than the SB group. The authors concluded that the DB procedure significantly improved knee stability although both techniques resulted in similar patient satisfaction as measured by outcome assessment (Table 3).

**Table 2:** Clinical study features

Study	Publication year	Type of study	Level of evidence
Wang et al. [28]	2004	Prospectively randomized study	II
Houe et al. [29]	2004	Retrospective comparative study	III
Fanelli et al. [30]	2008	Retrospective comparative study	V
Kim et al. [32]	2009	Retrospective comparative study	III
Shon OJ et al. [33]	2010	Retrospective comparative study	III
Yoon et al. [2]	2011	Randomized controlled study	II
Fanelli et al. [31]	2012	Retrospective comparative study	V
Li et al. [34]	2014	Lesser-quality randomized controlled study	II

**Table 3:** Characteristics of the included clinical studies

Study	No. of patients (S/D)	Graft choice(S/D)	Follow-up (mo) (S/D)
Wang et al. [28]	35(19/16)	Semitendinosus and gracilis	41.0/28.2
Houe et al. [29]	16(6/10)	Patellar tendon autograft/semitendinosus and gracilis autograft	31
Fanelli et al. [30]	90(45/45)	Achilles tendon and tibialis anterior allograft	≥24
Kim et al. [32]	29(G1: 8/ G2: 11/G3:10)	Achilles tendon allograft	32.4/31.9/33.6
Shon OJ et al. [33]	30(14/16)	Bone-patellar tendon-bone (BPTB) allograft and achilles tendon allograft/achilles tendon allograft	34/36
Yoon et al. [2]	53(25/28)	Achilles tendon allograft	28.5/27.4
Fanelli et al. [31]	90(45/45)	Achilles tendon and tibialis anterior allograft	≥24
Li et al. [34]	46(22/24)	Tibialis anterior allograft	25.1/23.5

*S* single bundle group, *D* double bundle group, *G1* group 1, single bundle, transtibial, *G2* group 2, single bundle, transtibial inlay technology, *G3* group 3, double bundle, transtibial inlay technology

## Discussion

### Biomechanical outcomes

#### AP stability

Race and Amis [16], Harner et al. [14], Markolf et al. [26, 27] and Wijdicks CA et al. [24] suggested that there is a statistically significant improvement in the AP stability of double-bundle reconstructions compared with single-bundle reconstructions. Conversely, the studies by Bergfeld et al. [25] and Mannor et al. [15] showed no significant improvement in AP stability with the addition of a second bundle.

The five studies suggesting improved AP stability with double-bundle reconstructions differ somewhat in graft characteristics and tensioning protocols. The study by Race and Amis [16] compared isolated tibial inlay 10-mm AL BPTB grafts with and without an additional 8-mm PM graft. The improved AP stability may be attributed to the improved stability resulting from the addition of a PM bundle; however, the effect may be due to the increased cross-sectional area of the double-bundle reconstructions rather than the specific location of the PM bundle. This same limitation applies to the work of Harner et al. [14] and Markolf et al. [26, 27] In their studies, Harner et al. [14] compared 10-mm AL Achilles tendon reconstructions with and without the addition of an 8-mm semitendinosus PM graft, whereas Markolf et al. [26, 27] compared 11-mm BPTB AL bundles with and without the addition of an 8-mm BPTB PM bundle.

Notably, Race and Amis [16] used a tensioning method to tighten the PM bundle in deep flexion (130°). This is consistent with recent data that suggests the importance of the PM bundle at higher degrees of flexion. This study tightened the AL bundle at 60° of flexion (magnitude based on the replication of the AP stability of the intact knee), whereas it is believed to be physiologically under more tension at greater flexion. This is particularly interesting because the failure to reproduce AP stability occurred only at flexion angles greater than 60°, suggesting that if tensioning was performed at deeper flexion, the single-bundle reconstruction may have successfully obtained intact AP stability.

Harner et al. [14] performed transtibial reconstructions and tensioned the grafts with a set tension (which did not restore AP stability to 90°) rather than using restoration of stability as an endpoint. Despite the improved stability single-bundle reconstructions have achieved, the stability of the intact knee has not been fully restored.

The work of Bergfeld et al. [25] conflicts with these five studies by showing no significant improvement in AP stability using tibial inlay Achilles tendon grafts of the same overall size in single- and double-bundle reconstructions.

Therefore, conflicting biomechanical studies with complex procedural differences do not show a definite advantage of double-bundle PCL reconstruction.

#### Graft tension

In addition to evaluating AP stability, several of the studies reviewed here measured graft tension in single- and double-bundle PCL reconstructions. Harner et al. [14] showed improved (increased) in situ graft forces in double-bundle reconstructions compared with single-bundle reconstructions. The same concerns mentioned previously with the tensioning protocol and transtibial fixation in this study should be taken into consideration in explaining this finding. This result is in direct opposition to the results of Markolf et al. [26, 27], who reported tension characteristics closer to the intact knee in single-bundle reconstructions rather than double-bundle reconstructions and only minor PCL tension changes with isolated PM bundle sectioning. Mannor et al. [15] suggested that altering the proximal/distal and anterior/posterior placement of a second bundle can create mirrored or reciprocal tension with increasing flexion. These contrasting outcomes reveal that many factors dictate the initial tension biomechanics of PCL reconstructions. However, the ideal tension and mode of failure remain elusive. It is unclear whether surgeons should strive to decrease tension to protect a graft from early failure and elongation or instead to replicate physiologic tension to protect secondary restraints that are subject to elongation over time. Other studies have shown that the initial graft tension in animal models do not reflect the graft tension measured at a later time.

Therefore, the paucity of research that investigates characteristics of the optimal graft tension hinders the possibility of determining the advantage of PCL double-bundle reconstructions.

### Clinical outcomes

#### Functional outcomes (functional assessment, functional score, or radiographic examination, subjective outcome, patient satisfaction)& AP stability

Seven published clinical comparisons did not show any statistically significant difference in AP stability between patients receiving single-bundle or double-bundle reconstructions for isolated PCL injury. However, Li Y et al. [34] argued that the DB group improved more than the SB group. Wang et al. [28], Kim et al. [32], Shon OJ et al. [33], Yoon KH et al. [2] and Li Y et al. [34] also found no difference in functional assessment, functional score, or radiographic examination. Houe and Jorgensen [29] described no difference in subjective outcome or patient satisfaction. Fanelli et al. [30, 31] have only published two abstracts without available detailed methodological and statistical data. All these studies are significantly limited by methodological concerns and the absence of a power analysis. The concerns include differential graft choices (patellar tendon [single] v hamstring [double]), very small PM bundle graft size, the tensioning protocol, the transtibial technique, among others.

Therefore, there is no published clinical evidence showing the superiority of functional outcomes after double-bundle PCL reconstructions.

## Limitation

Limitations of this systematic review resulted from the included studies. Low levels of evidence (some studies were Level III or IV) presented in this review exhibited the major limitations. Significant sources of selection bias were often present in the included studies, including heterogeneity in patient populations, surgical techniques, the type and size of grafts, the angles of knee flexion with graft fixation, and measures of clinical outcomes, which prevented a direct quantitative comparison between the two PCL reconstructions. Further studies are needed, including the biomechanical studies to address the concerns listed in this review and the well-designed clinical studies with appropriate surgical techniques and high-powered sample sizes. This would likely require a multicentre prospective cohort or randomized trial.

## Conclusions

This systematic review found that double-bundle reconstruction was significantly superior to single-bundle in biomechanical studies and clinical outcomes showed no significant differences between the two PCL reconstructions. However, there is limited evidence regarding the comparative advantages toward the optimal PCL reconstruction. The advancement of surgical techniques might be helpful for knee surgeons in making clinical decisions in PCL reconstruction.

## Abbreviations

PCL: Posterior cruciate ligament
AL: Anterolateral
PM: Posteromedial
SB: Single bundle
DB: Double bundle
PRISMA: Preferred Reporting Items for Systematic reviews and Meta-analyses
PLC: Posterolateral corner
AP: Anteroposterior
IKDC: International Knee Documentation Committee
BPTB: Bone Patellar Tendon Bone
